# Supplementary material for: Addressing the role of centromere sites in activation of ParB proteins for partition complex assembly
Source: PLoS One. 2020 May 7;15(5):e0226472. doi: 10.1371/journal.pone.0226472 (PMC7205306; doi:10.1371/journal.pone.0226472)
Supplement: S1 Fig — All transductions except the first involved cotransduction with a selective gene subsequently removed by FLP-mediated excision. Superscripts denote previously published strains (see Table 1). Lysogenization by λRS phages to integrate promoter-lacZ fusions has been described [40]. pcry denotes a cryptic promoter. (PPTX) [file pone.0226472.s001.pptx]

## Slide 1
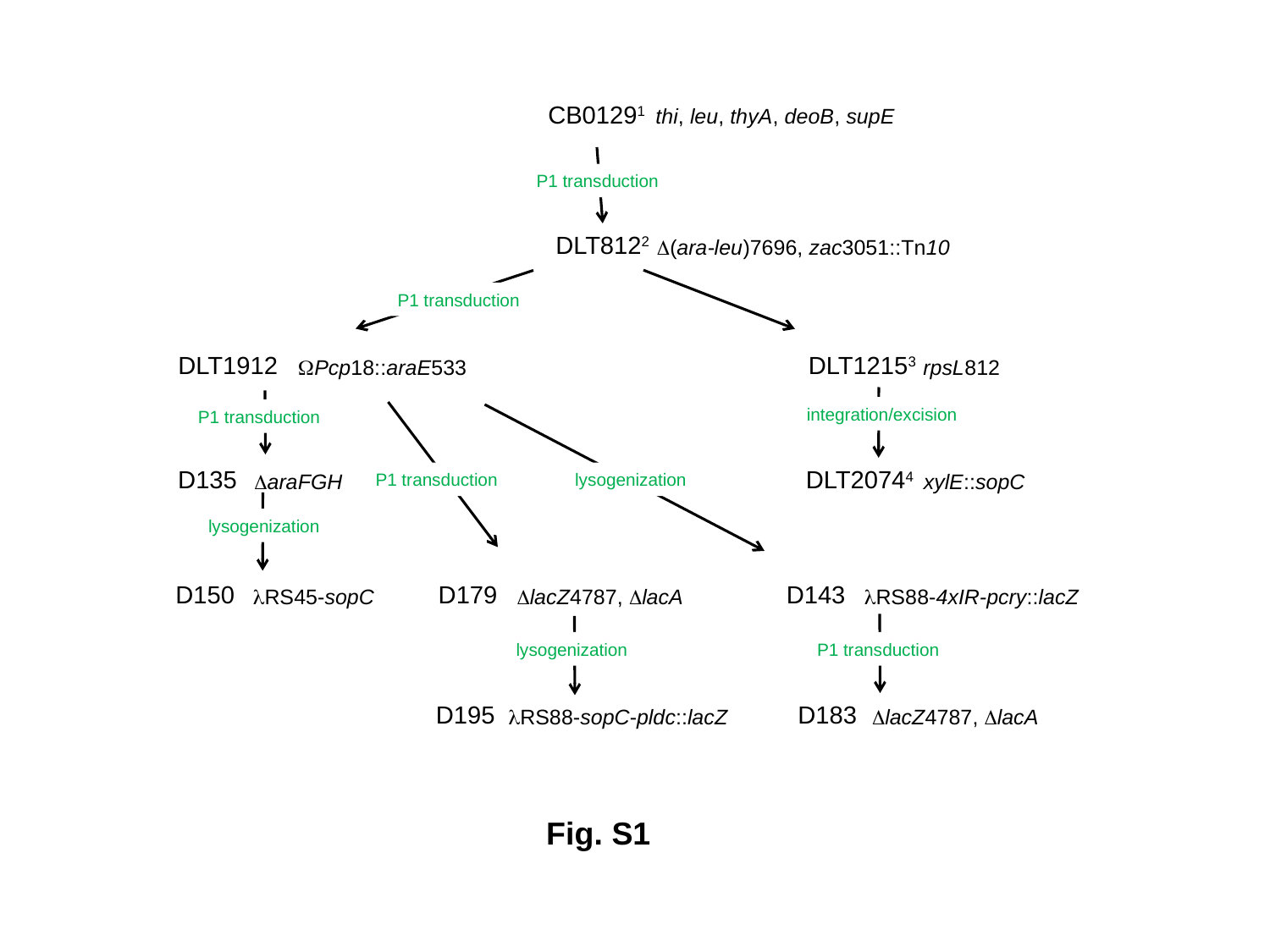

CB01291
thi, leu, thyA, deoB, supE
P1 transduction
DLT8122
D(ara-leu)7696, zac3051::Tn10
P1 transduction
DLT1912
WPcp18::araE533
DLT12153
rpsL812
integration/excision
P1 transduction
D135
DaraFGH
DLT20744
xylE::sopC
P1 transduction
lysogenization
lysogenization
D150
lRS45-sopC
D179
DlacZ4787, DlacA
D143
lRS88-4xIR-pcry::lacZ
lysogenization
P1 transduction
D195
lRS88-sopC-pldc::lacZ
D183
DlacZ4787, DlacA
Fig. S1
