# Supplementary figures and images for: Addressing the role of centromere sites in activation of ParB proteins for partition complex assembly
Source: PLoS One. 2020 May 7;15(5):e0226472. doi: 10.1371/journal.pone.0226472 (PMC7205306; doi:10.1371/journal.pone.0226472)

## Slide 1
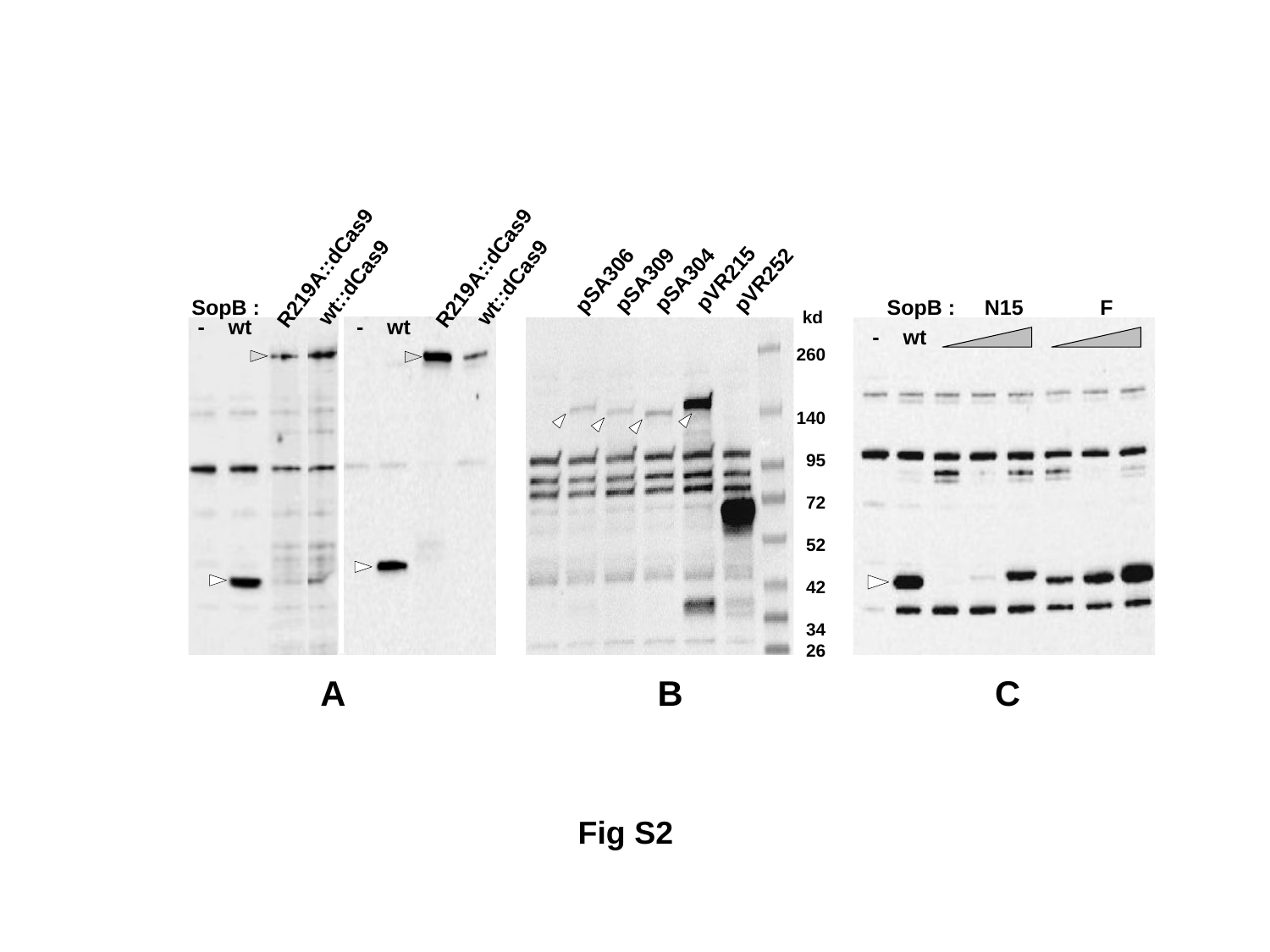

R219A::dCas9
R219A::dCas9
pVR215
pSA304
pSA309
pVR252
pSA306
wt::dCas9
wt::dCas9
SopB :
SopB : N15 F
kd
 - wt
 - wt
 - wt
260
140
 95
 72
 52
 42
 34
 26
A
B
C
Fig S2

Supplement: S2 Fig — A. Cells of strain DLT1215 and of derivatives carrying pDAG114 (wt mini-F; [59]), pCAT05 (sopBR219A::dcas9) and pCAT15 (sopB+dcas9), from cultures growing exponentially in MGC medium, Quantities of the R219A and wt SopB::dCas9 fusions (shaded arrowheads) relative to that of mini-F (~ 800 dimers/cell; clear arrowheads) were, respectively, 0.37 and 0.78 (left panel), and 1.1 and 0.20 (right panel), estimated using Image Lab (Biorad). Efficiency of SopB::dCas9 fusion protein transfer varied from one experiment to another: we show the results of two Western analyses of the same samples, which used MOPS buffer (left panel; irrelevant lanes between the third and fourth have been excised) and Tris-glycine (right panel) for electrophoresis and transfer. B. Exponential-phase cells of yeast strain W303 and derivatives harbouring plasmids that carry megfp::parB::tal fusions. The ladder shows prestained protein MW standards. Arrowed bands are, from left to right, those of fusions ParBRalstonia sp.::TaleU3M (formula MW 159kD), ParBS.pneumoniae::TaleoL1λ (153kD), ParBS.pneumoniae::TaleU3M (149kD), Or3::TaleU3M (157kD); the band (pVR252) is Or3::mEgfp (66kD) without a Tale. The first three fusions are produced from the pHIS3 promoter, the last two from the stronger pTEF promoter. C. As in A, except that instead of the SopB::dCas9 fusions, wt SopBN15 in strain D183/pNR189 and wt SopBF in D195/pDAG607 were analyzed. Arrowhead points to SopB from mini-F. Wedges represent graded arabinose inducer concentrations—0.1, 0.3, 1.0 nM and 0.03, 0.1, 0.3 nM respectively–used in the focus formation experiment of Fig 4. (PPTX) [file pone.0226472.s002.pptx]

## Slide 1
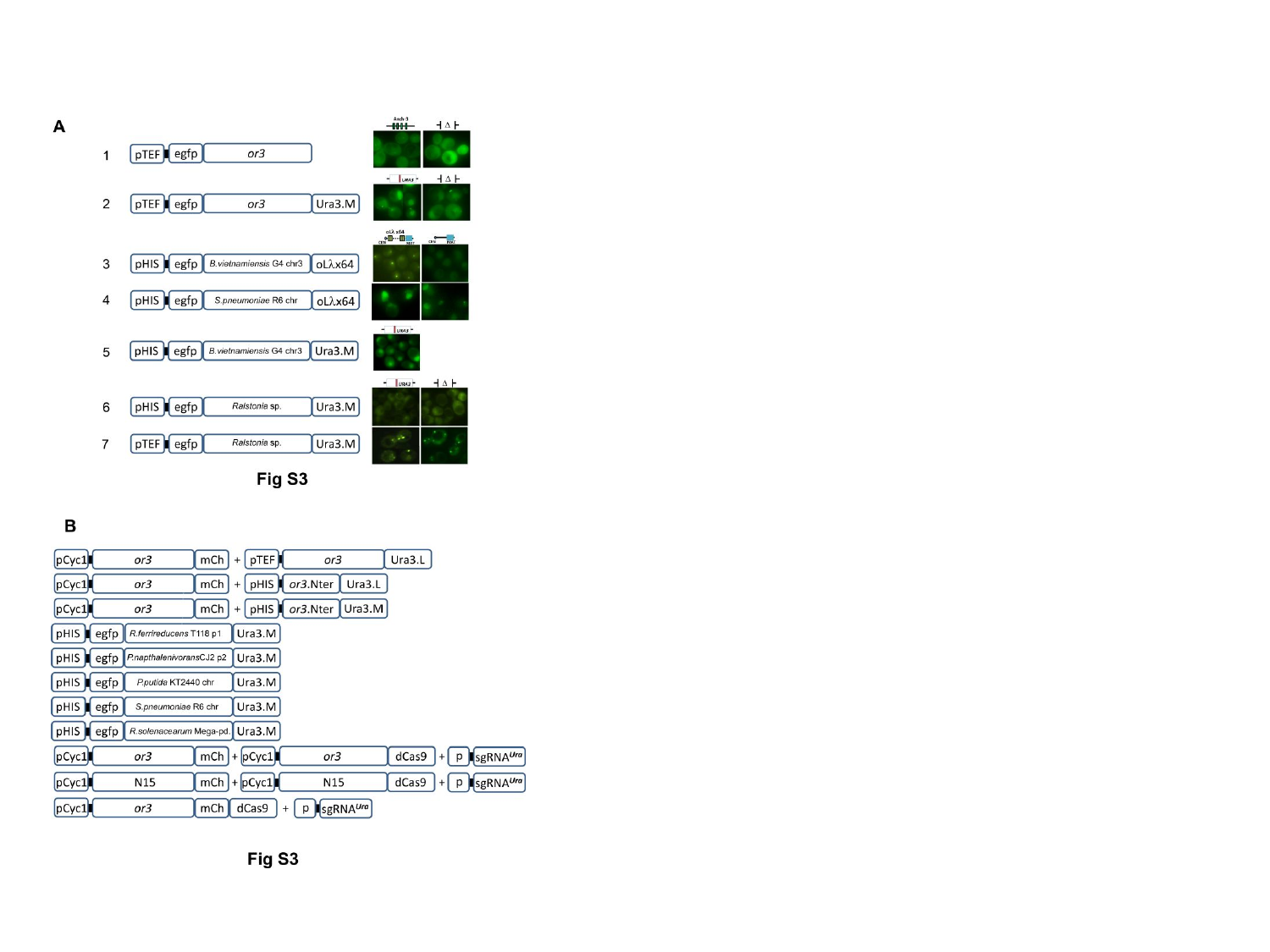

Supplement: S3 Fig — A. Examples: 1 –mEgfp::Or3 forms one focus per cell in the presence (left panel) but not in the absence (right panel) of an integrated Anch3 site; 2 –fusion of tal.U3M to mEgfp::Or3 results in addition of foci with or without the Tale target site, implying susceptibility of tripartite protein to aggregation; 3 –mEgfp::ParB::Tale.oLλ protein forms one strong focus per cell by simple, FROS-like binding in cells with a target site array (left), not in cells without (right), implying normal binding properties of tripartite protein; 4 –exchange of ParB unit in tripartite protein above results in occasional, target site-independent foci, implying functional incompatibility of the new ParB; 5 –exchange of Tale unit for tal.U3M does not result in new focus; 6 –tripartite protein produced from moderate-strength promoter frequently forms a single focus per cell (though independently of target site), but 7—when produced from a stronger promoter forms several foci both outside and inside nucleus, impying aggregation rather than partition complex assembly. B. Other configurations used in attempts to observe partition complex foci; none gave rise to single foci in cells carrying the Tale target sequence. (PPTX) [file pone.0226472.s003.pptx]
