# Supplementary material for: Addressing the role of centromere sites in activation of ParB proteins for partition complex assembly
Source: PLoS One. 2020 May 7;15(5):e0226472. doi: 10.1371/journal.pone.0226472 (PMC7205306; doi:10.1371/journal.pone.0226472)
Supplement: S4 Fig — A. Derivatives of strain D183 (4xIR—pcry::lacZ) carrying pNR189 (sopBN15; clear circles) or pNR197 (sopBN15::megfp; green circles) were grown exponentially for at least 10 generations in MGlyC with various concentrations of arabinose inducer, and culture samples assayed for β-galactosidase activity. Specific activity in the absence of arabinose was 17 Miller units. B. Strain D195 (sopC—pldc::lacZ) carrying pDAG607 (sopBF) and pNR198 (sopBF.R219A) was grown and assayed as in A. Specific activity without arabinose was 525 MU. C: D195 carrying pDAG525 (sopBF::megfp) was grown and assayed as in A with various concentrations of anhydrotetracycline. Specific activity without arabinose was 436 MU. (PPTX) [file pone.0226472.s004.pptx]

## Slide 1
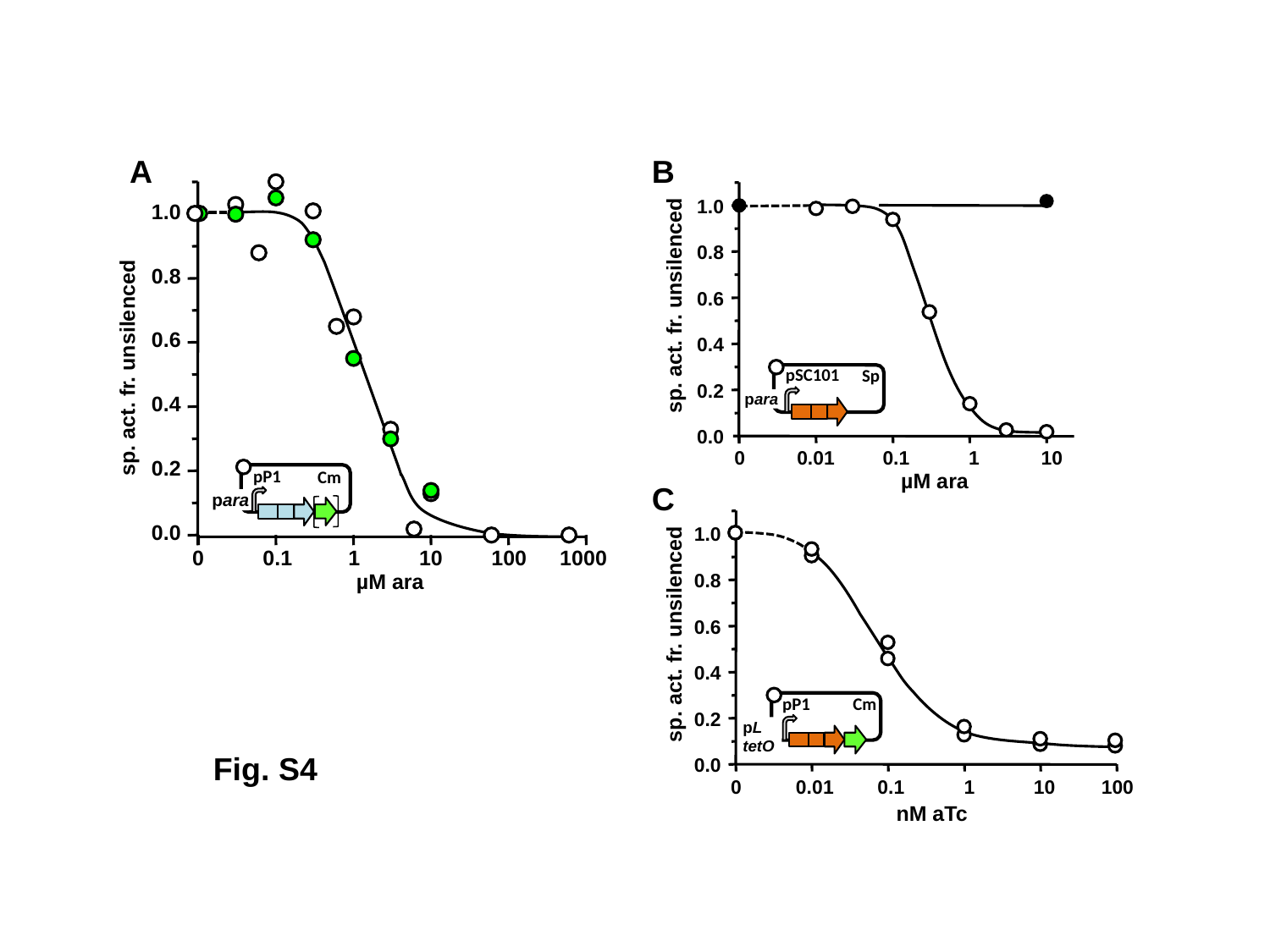

A
 B
1.0
0.8
0.6
sp. act. fr. unsilenced
0.4
0.2
pP1
Cm
para
0.0
0
0.1
1
10
100
1000
µM ara
1.0
0.8
0.6
sp. act. fr. unsilenced
0.4
pSC101
Sp
para
0.2
0.0
0
0.01
0.1
1
10
µM ara
 C
1.0
0.8
0.6
sp. act. fr. unsilenced
0.4
pP1
Cm
pL
tetO
0.2
0.0
0
0.01
0.1
1
10
100
nM aTc
Fig. S4
