# Supplementary material for: Addressing the role of centromere sites in activation of ParB proteins for partition complex assembly
Source: PLoS One. 2020 May 7;15(5):e0226472. doi: 10.1371/journal.pone.0226472 (PMC7205306; doi:10.1371/journal.pone.0226472)
Supplement: S1 Table — Strains carrying chromosomal xylE and sopC sequences on the chromosome and genes for the corresponding sgRNAs on an expression vector were transformed with plasmids from which production of the SopB::dCas9 fusion or the equivalent active Cas9 fusion protein could be induced. Viability of transformants on agar medium was scored (see Materials & methods). (DOCX) [file pone.0226472.s005.docx]

| Strain | colony formation | |
| --- | --- | --- |
|  | pCAT02 (pLtetO::dcas9) | pCAT13 (pLtetO::cas9+) |
| DLT1215 : |  |  |
| pCAT06 (sgRNA-0) | + | + |
| pCAT184 (sgRNA-xylE) | + | - |
| pCAT08 (sgRNA-sopC) | + | + |
| DLT2074 : |  |  |
| pCAT06 (sgRNA-0) | + | + |
| pCAT184 (sgRNA-xylE) | + | - |
| pCAT08 (sgRNA-sopC) | + | - |

Table S1 Interaction of target sequences with Cas9 guide RNAs.
