# Supplementary material for: Addressing the role of centromere sites in activation of ParB proteins for partition complex assembly
Source: PLoS One. 2020 May 7;15(5):e0226472. doi: 10.1371/journal.pone.0226472 (PMC7205306; doi:10.1371/journal.pone.0226472)
Supplement: Attachment — Submitted filename: Response to Reviewers.docx [file pone.0226472.s006.docx]

Dear Dr Rybenkov

We have considered the the reviewers' comments and your own concerning our paper "Role of centromere sites in activation of ParB proteins for partition complex assembly", PONE-D-19-33138.

Before detailing our responses I should point out that the original MS included two errors :

First, the version of Fig 1 submitted is not the one that corresponds to the Fig 1 legend or to the text ; the portions Bi and Biii were exchanged. This might have contributed to difficulties in following the logic of the paper. The version resubmitted has been corrected.

Second, the description of the construction of a series of plasmids, under "SopB::mVenus visualization" was far from complete. This has now been corrected, see p.7.

Other points :

Addition of two new supplementary figures and renumbering to preserve the correct order.

A number of minor corrections and modifications, not worth listing but readily seen in the marked up file.

The revisions necessitated addition of three more references (numbers 48, 58 and 60 in the new MS). We have also added a fourth (number 37, Osorio-Valeriano et al) because it represents the same major advance as the Soh et al paper already cited but had not come out at the time of our original submission.

**Editor's comments :**

1 - *Specifically, please include additional characterization of your system to substantiate your claim that the failure of chimeric ParB to produce foci was indeed due to its inability to bind ParS and not due to other uninteresting problems with construction of chimeric proteins.*

Reply:	By "inability to bind parS" we presume you mean "inability to spread in the absence of parS", since this is our main conclusion. We agree that difficulties in production and behaviour of the chimeric proteins could limit the certainty of this conclusion. We have added Western blot data (Fig S2), as also suggested by Reviewer 1, to demonstrate normal levels of chimeric protein production, and a sentence to point this out (top p.16). Although in several cases (Figs 4C, 5A, S3A, ...) the chimeric proteins are still capable of binding parS, whereas in others we cannot tell whether they do or not owing to apparent abnormal behaviour (your "uninteresting problems" presumably) of the large chimeras. This was acknowledged in the original MS on p.15 following Fig. 3 legend "... formation of aggregates rather than bona fide foci ... " and is evident in some of the examples we now provide in Fig S3. We have also underlined this phenomenon, and thus softened our conclusion, with a modified sentence (the third) of the Discussion. In fact it is exactly these limits to our conclusions of the large protein fusion experiments that lead to and justify the hybrid SopB protein experiments in which centromere binding is demonstrably not a problem and which buttress our conclusion that centromere binding is needed for focus formation.

*2 - Reviewer 1 suggested a number of important controls to this end. I would also recommend a positive control, when you join the N- and C-terminal domains of ParB via the same linker that you use in other chimera and observe ParS-dependent formation of the foci.*

Reply:	It is not clear what new constructions are being suggested. Our general experience with joining ParB proteins to Gfp-type peptides, Nter or Cter (refs 3-5 in the MS), is that focus formation is achieved with a wide variety of linker peptides. If the order of the Nter and Cter domains of the N15-F hybrid is in question, we know (but saw no need to say) that only the hybrid used in the paper is functional, and that the equivalent F-N15 hybrid is not. If the ParB::Cas9 and ParB::Tale fusions are the point, we made no systematic attempt to test variations of linker peptide sequence or length, particularly as Tale::ParB fusions proved far more difficult to make and maintain (none appear in our Fig S3 summary) than ParB::Tale fusions, regardless of linker sequence.

*3 - There is also a logical gap between your observations and the conclusion as pointed out by the Reviewer 2. It is really impossible to draw such a strong conclusion as you propose from a negative result.*

Reply:	We do not agree that there is a logical gap; Reviewer 2 certainly does not explain it (see comment below). We propose certain possibilities (Fig 1), test them in the experiments subsequently described, and find that only one of the possibilities is not ruled out by the results. We do not claim that this is a strong conclusion, rather that it is the only one left standing, as must be the case in the absence of a positive demonstration. The very recent provision of such a demonstration (Soh et al, Osorio et al) reinforces our conclusion but we do not pretend that this alters its essentially provisional nature.

*4 - Please reword the title of your manuscript to better reflect your observations and the revise the text of the manuscript accordingly.*

Reply:	The title is entirely neutral. It does not say in advance what we conclude the role of centromere sites to be, or even that there necessarily is one. If one wishes to look behind its literal meaning, the title implies that centromere sites have already been proposed to have a role in ParB activation, as Reviewer 2 points out, and this study is aimed at defining that role. We do not understand why Reviewer 2 or you would object to this.

*5 - 2. We note you have included a table to which you do not refer in the text of your manuscript. Please ensure that you refer to Table 4 in your text; if accepted, production will need this reference to link the reader to the Table.*

Reply:	We have now included reference to Table 4, under "Plasmid constructions" on p.6.

**Reviewer #1:**

*The figures already include several controls that show that the experiments are working as intended. However, two (hopefully easy) control experiments should be added to make sure that the negative results are not caused by obvious experimental problems.*

*A) Can the expression levels of tagged and untagged ParB variants be quantified? Semi-quantitative Western blotting may be sufficient to show that all proteins are present in reasonable amounts.*

We have included Western blot data as Fig. S2. They show that for the three experimental situations examined the amounts of full-size ParB fusion protein are at least at the levels at which the corresponding wild type ParB proteins would form partition complexes. In the case of the SopB fusion proteins this conclusion is reached by comparison with wt SopB. In the case of the Gfp::ParB::Tale fusions it is relative to visible fluorescence levels; Fig S2B shows that the full-sized fusions are the only significant anti-Gfp species present.

*B) All experiments rely on the specific binding of an engineered or mutant protein (dCas9 or TALEN fusion proteins and SopB R219A mutants) to a target sequence. The efficiency of the specific binding is however not directly tested for any of the presented approaches. Cell killing by Cas9 implies target sequence recognition. However, very transient or rare binding might be sufficient for cell killing, while ParB nucleation might require more stable binding. Can the localization of these proteins be demonstrated directly by ChIP-sequencing or ChIP-qPCR?*

In vivo binding of dCas9 is generally rather stable (occupancy >30 mins E.coli, Jones et al Science 357, 1420-1424; >3hrs eucaryotes, Ma et al J. Cell Biol. 214, 529-537), though it is possible that at the sites used transient binding of a dCas9-parB chimera is insufficient to trigger nucleation. We have added a sentence to the start of the Discussion to cover this possibility. But whether durable or transient, binding did not enable nucleation, which was the aim of creating the chimeric proteins in the first place.
In any case ChiP is not a method of choice for measuring efficiency of binding of a single protein. It can report the presence of proteins at a given DNA locus above background, as in its successful use to demonstrate ParB::GFP spreading in yeast (Saad) and SopB::mVenus in bacteria (Sanchez). Without amplification by spreading, variation in antibody specificity and PCR efficiency makes detection of even tight binding of a single molecule unreliable. Restriction site protection would be a possibility but given the unlikelihood of brief occupancy being the problem we did not consider the investment worthwhile.

*Local enrichment requires energy input. Simple uncoupling of CTP binding/hydrolysis from target sequence recognition (as alluded to at the end of the discussion) will accordingly disrupt ParB accumulation even at heterologous sites. Successful artificial targeting should require mechanistic linkage of CTP binding/hydrolysis with the (then alternative) DNA binding of the fusion partner (dCas9 or TALEN). The statement should thus be softened.*

The reviewer appears to propose something other than what we are suggesting at the end of the discussion. It is hard to see how the alternative binding partner could be made to transmit the appropriate signal to the fused ParB. In any case it is the parB we would mutate, in the hope that the low energy barrier to CTP-induced transition already suggested by Soh et al's data (Fig. 3C) could be further lowered to the point where spontaneous transition to active conformer state replaces parS-mediated stimulation often enough to enable a focus to become visible at the alternative binding site.

We considered inserting this suggestion into the final figure recommended by the reviewer (see next) but given that isolation of an appropriate ParB mutant is entirely speculative we decided such an addition premature.

*Fig. 1 (or a final figure) should include the recently proposed DNA spreading model.*

The recent model is not fundamentally different from Biii, rather it is extended and far more concrete, so addition to Fig. 1 is not appropriate. We have followed the reviewer's suggestion to add a final figure, Fig. 6, that stresses the relationship between the two, hoping that this is what the reviewer had in mind. A few words added at the bottom of p.21 refer to it.

*A short introduction of the N15 prophage might be helpful for the less experienced reader.*

We have added a sentence at the bottom of p.16 to fill this gap.

*Page 15, 2nd paragraphs: Please provide a list of all conditions tested for targeting (different ParBs, tripartite fusion…). Even without full documentation, the information may be useful to some readers.*

We have added a supplementary figure (Fig. S3) in two parts, one (A) with images illustrating particular points (parS binding, Tale target binding, interference by aggregates), the other (B) as a list of constructions tried and tested.

*Fig. 3: The ParB foci in the yeast images are quite faint. Please provide arrowheads or enhanced contrast.*

The problem here was in the conversion to .pdf images. The final on-line version should be much clearer.

**Reviewer #2:**

*At current level, the hypothesis raised in this work was denied by the experiment result, and no new knowledge was created.*

We concede, obviously, that the result was negative in the sense that no evidence was obtained for purely ParB-ParB interaction as a basis for spreading, but this is not the same as "no new knowledge". The essential message is "if you are thinking about trying centromere-independent spreading, think again, it has already been tried."

*The conclusion, parB proteins need their cognate centromeres to become capable of spreading, has been extensively approved in various species.*

There is a distinction to be made here, as we explain in the Introduction (bottom, p.3) : does the need for parS in spreading consist of activating ParB to enable it, or of providing a focal point to concentrate ParB molecules which are activated by other means ? No previous study has explicitly attempted to discriminate between these two options. Several authors have suggested direct parS-mediated activation but this was opened to doubt by other observations (Surtees & Funnell; Hyde et al), as detailed in the Introduction. The reviewer goes with the common assumption but this is not the same as a demonstration. In fact it is only the very recent report by Soh et al that puts the issue beyond reasonable doubt. Our conclusion is consistent with it.

*This is not the conclusion from this work.*

If we understand the reviewer, this is exactly the conclusion of this work. It is just that we have also sought to examine other possible mechanisms.

*On the other hand, the context structure, logical connection and details of the language of this manuscript need improving to reach the publish level.*

The reviewer does not explain what problems he sees with "the context structure" and "logical connection", so there seems nothing to reply to. Likewise, what the reviewer means by improvement in "the language of this manuscript" is not explained.
