# Supplementary material for: Addressing the role of centromere sites in activation of ParB proteins for partition complex assembly
Source: PLoS One. 2020 May 7;15(5):e0226472. doi: 10.1371/journal.pone.0226472 (PMC7205306; doi:10.1371/journal.pone.0226472)
Supplement: Attachment — Submitted filename: Responses to editor & reviewer.docx [file pone.0226472.s007.docx]

Editor:

*Specifically, I ask you again to re-evaluate your conclusions, which at times appear to reach further than warranted by the data. This is a classic case of when a failure to prove a phenomenon does not prove its absence. I agree with the reviewer that your findings carry a constructive message. However, by overinterpreting them, you negate their impact. Please note that one of PLOS ONE publication criteria states: "Conclusions are presented in an appropriate fashion and are supported by the data". In my view, this means that conclusions must be clearly separated from inferences.*

Reply:	We can only agree with the principle at issue here, though apparently you consider our attempts to deal with it in our first revision (between [ ] below) inadequate. It would have been helpful if you could have provided a clearer indication of what you mean by "overinterpretation", using one or two specific examples from the text for instance. We can only suppose that you see overinterpretation in either or both of two ways : (i) any attempt to discuss the results beyond the simple observation of lack of focus formation by hybrid ParB proteins is unjustified ; (ii) we have been too assertive in summary or linking sentences – "concluding" rather than "inferring", for example.

If your objection is the former, we would reply as follows. The major conclusion – that ParB proteins do not spread in the absence of their centromeres – is clearly provisional, being based in this paper only on the proteins tested and our experimental approach (one can hardly be expected to test every conceivable configuration). It does however correspond to the general assumption in the field (as seen by the attitude of reviewer 2). But once it is provisionally accepted the issue becomes what the centromere is needed for, which is why we discuss (initially in the 2^nd^, now the 3^rd^ paragraph of the Discussion) the options outlined in Fig 1 and how our results bear on them, leading ineluctably to centromere activation of ParB spreading. If this is not suitable for discussion then not only does the Discussion lose most of its interest but we would have fallen far short of "Addressing" (as the reviewer recommends we modify the title) the topic. It would also render superfluous both the fuller perspective provided by Fig 1and the Fig 6 recommended by the reviewer.

If your objection is our manner of expression then you may have a point : readers might well get the impression of stronger finality in our statements than we intended to convey. On this assumption, we have modified the text at various points, including the Abstract, as shown in the marked up copy. In particular, we have modified the start of the Discussion to squarely explain the limits of our interpretation, and on p.20 emphasized the provisional nature of the deduced centromere role.

[1 - *Specifically, please include additional characterization of your system to substantiate your claim that the failure of chimeric ParB to produce foci was indeed due to its inability to bind ParS and not due to other uninteresting problems with construction of chimeric proteins.*

Reply:	By "inability to bind parS" we presume you mean "inability to spread in the absence of parS", since this is our main conclusion. We agree that difficulties in production and behaviour of the chimeric proteins could limit the certainty of this conclusion. We have added Western blot data (Fig S2), as also suggested by Reviewer 1, to demonstrate normal levels of chimeric protein production, and a sentence to point this out (top p.16). Although in several cases (Figs 4C, 5A, S3A, ...) the chimeric proteins are still capable of binding parS, whereas in others we cannot tell whether they do or not owing to apparent abnormal behaviour (your "uninteresting problems" presumably) of the large chimeras. This was acknowledged in the original MS on p.15 following Fig. 3 legend "... formation of aggregates rather than bona fide foci ... " and is evident in some of the examples we now provide in Fig S3. We have also underlined this phenomenon, and thus softened our conclusion, with a modified sentence (the third) of the Discussion. In fact it is exactly these limits to our conclusions of the large protein fusion experiments that lead to and justify the hybrid SopB protein experiments in which centromere binding is demonstrably not a problem and which buttress our conclusion that centromere binding is needed for focus formation.]

Reviewer #1:

The authors have made several modifications to the manuscript text and added control experiments in response to the reviewers’ and editor’s comments. They have seriously considered the comments and have made appropriate changes or alternatively provided explanations in the written response to the comments.

While the outcomes remain negative, the manuscript with its well-executed experiments will be a valuable addition to the literature, at the least instructing other researchers when attempting similar experiments in the future. Altogether, this reviewer recommends publication of the work in its current form. The authors may however consider the points given below prior to publication.

Minor points:

*Following the comments from the other reviewer and the editor, the title of the manuscript could be slightly toned down to ‘Addressing the role of centromeric sites in activation of ParB proteins for partition complex assembly’.*

Reply:	We have adopted the title suggested.

*Throughout the manuscript the involvement of ParB oligomerization in ParB spreading is implicated. However, the conclusions of this work are largely independent thereof. Thus, the authors may want to consider making their work stand regardless of ParB oligomerization by eliminating such statements.*

Reply:	Oligomerization via N-terminal domains of ParB dimers has been a regular if seldom demonstrated part of ParB research for 20 years, so we think it is appropriate to refer to it in the Introduction. But as the the reviewer points out, our results do not bear directly on oligomerization itself . And the recent model of Soh et al scarcely involves it. So from Results on we have referred simply to "N-terminal domain".

*The figure legends are rather tough to read, mainly due to the listing of many genotypes. Information on genotypes could be removed from the legends altogether. The information is largely provided in the figure panels. Only strain names and plasmid names could be included in the figure legends or alternatively the use of plasmids and strains including genotypes for each figure could be listed in a separate table.*

Reply:	We have removed plasmid genotypes from the legends to Figs 2, 4 and 5, leaving only strain and plasmid names.

*The figure legend to Figure S4 is particularly long. Please consider moving information on the Western blot protocol to the methods section.*

Reply:	The reviewer means Fig S2. As suggested, the purely protocol portions have been transposed to a new Western blotting section in Materials & Methods.

*Panel designators are not consistently noted throughout the figure legends. A : in Figure 4. A- and (A) in Figure 3. A – in Figure 1. Please revise.*

Reply:	Designations have been corrected for all legends, including those for Supplementary figures to the format A. .
